# Pregnancy after the diagnosis of lymphangioleiomyomatosis (LAM)

**DOI:** 10.1186/s13023-021-01776-7

**Published:** 2021-03-17

**Authors:** Lisha Shen, Whenshuai Xu, Jinsong Gao, Jun Wang, Jiannan Huang, Yani Wang, Yudi He, Yanli Yang, Xinlun Tian, Kai-Feng Xu

**Affiliations:** 1 Department of Pulmonary and Critical Care Medicine, State Key Laboratory of Complex Severe and Rare Diseases, Peking Union Medical College Hospital, Chinese Academy of Medical Sciences, Beijing, China; 2 Department of Respiratory Disease, The First Affiliated Hospital, Zhejiang University School of Medicine, Hangzhou, China; 3 Department of Obstetrics and Gynecology, Peking Union Medical College Hospital, Chinese Academy of Medical Sciences, Beijing, China

**Keywords:** Lymphangioleiomyomatosis, Tuberous sclerosis complex, Pregnancy, Sirolimus, Pneumothorax

## Abstract

**Background:**

Lymphangioleiomyomatosis (LAM) is a rare disease that almost exclusively affects women of reproductive age. Patients are warned of the increased risks if they become pregnant. However, information on pregnancy in patients after the diagnosis of LAM is limited.

**Methods:**

Patients were collected from the LAM registry study at Peking Union Medical College Hospital, Beijing, China. Patients with a history of pregnancy after the diagnosis of LAM were included. Medical records were reviewed, and baseline information and data during and after pregnancy were collected in May 2018.

**Results:**

Thirty patients with a total of 34 pregnancies after the diagnosis of LAM were included. Livebirth, spontaneous abortion and induced abortion occurred in 10, 6 and 18 pregnancies, respectively. Sirolimus treatment was common (17/34). A total of 6/10, 5/6, and 6/18 patients with livebirths, spontaneous abortions, and induced abortions respectively, had a history of sirolimus treatment. Ten pregnancies (29.4%) had LAM-associated complications during pregnancy, including the exacerbation of dyspnea in 7 patients, pneumothorax in 3 patients (2 resulting in induced abortion and 1 successful parturition), and spontaneous bleeding of renal angiomyolipomas in 2 patients (both having successful parturition). No chylothorax was found during pregnancy. There were six pregnancies in six patients (17.6%) who had a history of livebirth after sirolimus treatment for LAM (all having successful parturition and healthy infants); two of these patients reported exacerbated dyspnea after parturition compared with before pregnancy.

**Conclusions:**

Patients with LAM, especially those taking sirolimus before pregnancy, were at a higher risk of spontaneous abortion. Complications such as pneumothorax, bleeding of renal angiomyolipoma, and exacerbated dyspnea during pregnancy were common. In patients without spontaneous abortion, sirolimus discontinuation before or during pregnancy did not lead to increased adverse neonatal outcomes.

## Introduction

Lymphangioleiomyomatosis (LAM) is a rare disease that mostly afflicts women and primarily affects the lung, kidney, and lymphatic system [1]. The prevalence of LAM varies, ranging from 1 to 9 per million women in the general population and from 30 to 40% in women with tuberous sclerosis complex (TSC) [2–4]. Patients with LAM suffer from worsening dyspnea and an increasing number of cysts by computed tomography (CT) scan.

Since patients are mostly diagnosed with LAM during their childbearing years, pregnancy has become an important issue for these patients. The European Respiratory Society (ERS) guidelines for LAM mentioned that it is likely that pregnancy in patients with LAM is associated with an increased risk of pneumothorax, chylothorax, bleeding from angiomyolipoma and an acceleration of lung function decline [5]. The guidelines recommended that the decision to become pregnant should be made on an individual basis and that patients who have severe disease should be discouraged [5]. Another issue is the safety of sirolimus use before and during pregnancy. Current recommendations from the manufacturer of the drug suggest discontinuing sirolimus 12 weeks before pregnancy. Such a recommendation may potentially carry the risk of disease progression after discontinuing sirolimus.

In the current research, we investigated the pregnancy outcomes after a diagnosis of LAM, with a specific focus on patients who used sirolimus before or during pregnancy.

## Materials and methods

### Study population

Patients were from the LAM registry study of Peking Union Medical College Hospital, Beijing, China between June 2011 and May 2018. Pregnancy questionnaires were sent to all patients in the registry in May 2018, and telephone interviews were conducted with some patients to clarify and ensure accurate information. Medical records regarding pregnancy, LAM diagnosis, and use of sirolimus were reviewed. Available cases were reevaluated for the diagnosis of LAM based on the American Thoracic Society (ATS) and Japanese Respiratory Society (JRS) guidelines published in 2017 [6]. The inclusion criteria included (1) definite diagnosis of LAM, (2) probable diagnosis of LAM if it was not a definite diagnosis based on the guidelines of the ATS [5], and (3) pregnancy after the diagnosis of LAM. Subjects were excluded if the diagnosis of LAM was not confirmed. The protocol for this study was approved by the Ethical Committee of Peking Union Medical College Hospital (S-K1246).

### Materials

The following information was collected. (1) Baseline data were defined as the latest follow-up reports before pregnancy and after the diagnosis of LAM. (2) LAM-related symptoms before, during and after pregnancy were collected. Data before pregnancy were defined as the last report of each test we could obtain before pregnancy and after the diagnosis of LAM. Data after pregnancy were defined as the first report of every test we could obtain after the termination of pregnancy. Pneumothorax was confirmed by radiologic imaging of the chest. Chylothorax was confirmed by radiologic imaging and pleural fluid analysis. Spontaneous bleeding of angiomyolipoma (AML) was supported by clinical manifestations and any kind of imaging, including ultrasound, computed tomography, or magnetic resonance. (3) Detailed information on sirolimus usage was recorded if patients took sirolimus before pregnancy. (4) The outcome of pregnancy and any abnormalities of pregnancy were collected.

### Statistical analysis

Because of the small number of patients involved in this observational study, a description of cases was used instead of a statistical analysis.

## Results

### Patient characteristics

**Fig. 1:**
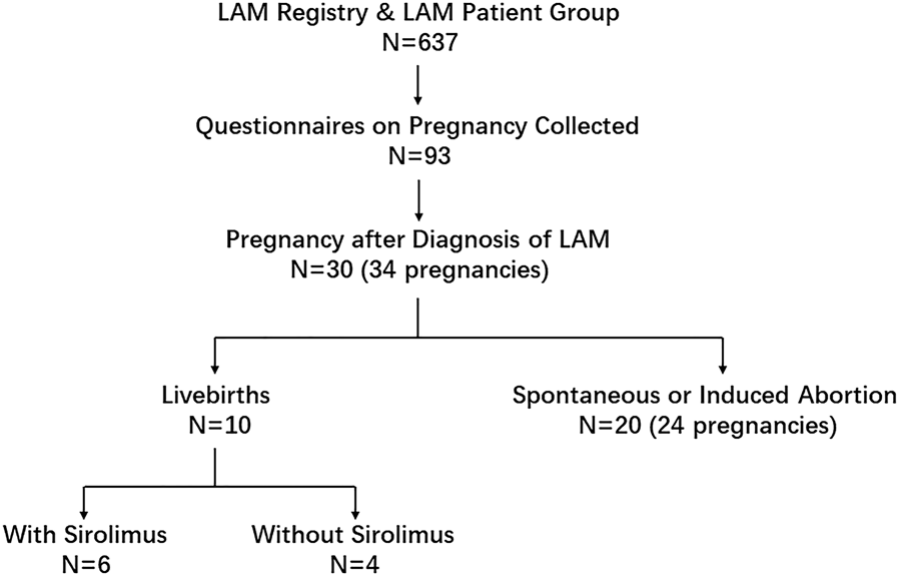
Study flowchart

Ninety-three valid questionnaires were collected. After reviewing the data, 30 patients with 34 pregnancies were included in the study according to the inclusion and exclusion criteria (Fig. 1). Among them, 28 patients were diagnosed with definite LAM, while the other 2 patients were diagnosed with probable LAM. The two patients with probable LAM had characteristic CT features of LAM without other supporting evidence or vascular endothelial growth factor-D results.

The baseline clinical features of these patients are shown in Table 1. Most of them were patients with sporadic LAM. Pneumothorax was the most common complication, followed by renal AML and chylothorax. Pulmonary function tests showed mildly damaged ventilatory function and moderately damaged diffusion function. Eighteen patients had 32 pregnancies before the diagnosis of LAM. Sixteen of them had 20 livebirths, with a livebirth rate of 62.5% (20/32). The other 12 pregnancies were terminated by induced abortion without any spontaneous abortion. Two patients reported pneumothorax 5 and 2 times, respectively, during pregnancy, and both delivered the infants successfully.

**Table 1:** Baseline characteristics of patients with lymphangioleiomyomatosis

Characteristics	Numbers (%)
Patients	30
Age of diagnosis (years)	29.4 ± 4.9
Diagnosis	
TSC-LAM	3 (10.0)
Sporadic-LAM	27 (90.0)
Complications	
Renal AML	11 (36.7)
History of pneumothorax	14 (56.7)
History of chylothorax	7 (23.3)
Pulmonary function testing^a^	
FEV_1_ (L)	2.17 ± 0.77
FEV_1_/pred (%)	73.78 ± 25.66
FVC (L)	3.19 ± 0.57
FVC/pred (%)	93.70 ± 16.58
FEV_1_/FVC (%)	66.80 ± 19.19
DLco (mmol/min/kPa)	5.27 ± 2.29
DLco pred (%)	48.31 ± 21.13
6-min walking distance (m)^b^	509.43 ± 68.90
Total score on St. George’s Respiratory Questionnaire^c^	32.91 ± 19.17
History of pregnancy before diagnosis of LAM	18 (60.0)
Patients with live birth	16 (53.3)
Patients with spontaneous abortion	0
Patients with pneumothorax during pregnancy	2 (6.7)

Data with a normal distribution are presented as the means ± standard deviation (SD), and data with a nonnormal distribution are reported as the median (Q1, Q3)

*AML* angiomyolipoma, *DLco* diffusing capacity for carbon monoxide, *FEV*_*1*_ forced expiratory volume in 1 s, *FVC* forced vital capacity, *LAM* lymphangioleiomyomatosis, *TSC* tuberous sclerosis complex

^a^Sample size for pulmonary function testing was 21

^b^Sample size for 6-min walking testing was 21

^c^Sample size for St. George’s respiratory questionnaire was 22

### Pregnancy outcomes

**Fig. 2:**
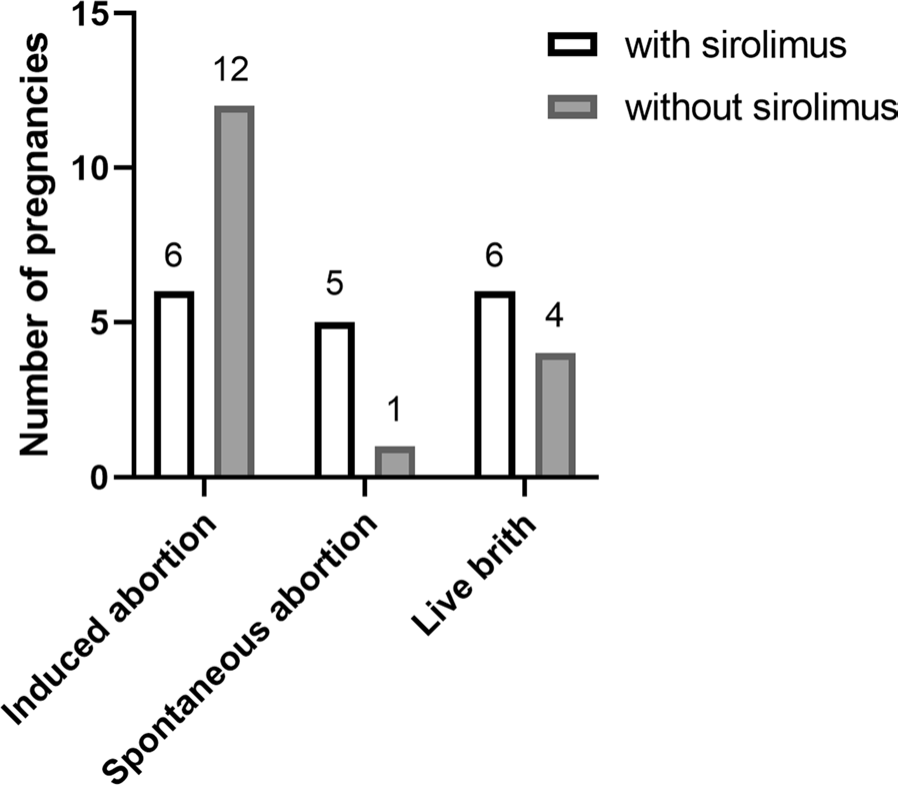
Pregnancy outcomes of patients after the diagnosis of lymphangioleiomyomatosis Sirolimus history before pregnancy for livebirths, spontaneous abortions and induced abortions were noted

Thirty patients had a total of 34 pregnancies after the diagnosis of LAM. Of the 34 pregnancies, 17 occurred in patients who had never taken sirolimus before pregnancy, and the other 17 occurred in patients who had. The outcomes of all the pregnancies are shown in Fig. 2. Eighteen pregnancies were terminated artificially because of pneumothorax (n = 2), sirolimus history (n = 6) or worries of worsening symptoms or complications of LAM during pregnancy. Only 10 pregnancies resulted in successful full-term livebirths; for 6 of these, the patient had sirolimus intake. Among those 10 patients who developed full-term livebirths, 6 had a history of pregnancy before diagnosis of LAM (4 livebirths, 2 induced abortions).

Among six patients who experienced spontaneous abortions, 5 had a history of sirolimus intake before pregnancy. All 5 patients were on sirolimus when they were pregnant, and the duration of sirolimus usage ranged from 4 to 50 months before pregnancy. Four patients were on a dose of 1 mg per day, while 1 patient was taking 2 mg per day. Four of them had at least one LAM-associated complication before this pregnancy. Four of the 5 patients had histories of pregnancy and livebirth before this pregnancy, while one patient had never been pregnant. Four pregnancies lasted for 6 weeks until spontaneous abortion, and 1 lasted for only 2 weeks.

### Complications during pregnancy

Complications during pregnancy were reported in 10 patients. Seven patients reported exacerbation of dyspnea. Two of them had induced abortion in the first trimester, and the remaining 5 had successful deliveries. Five patients received oxygen inhalation (duration ranging from 1 to 24 h per day). Six patients reported recovering from dyspnea after termination of the pregnancy, and 1 patient reported mild aggravation of dyspnea compared with before pregnancy.

Three patients reported pneumothorax (including one who also reported exacerbation of dyspnea). In one patient, bilateral pneumothorax was found at week 22 of pregnancy, and abortion was induced. The second patient who had recurrent pneumothorax was found to be pregnant when she was receiving drainage for a pneumothorax, and abortion was induced in her first trimester. The third patient had pneumothorax at week 28 and finally delivered a full-term infant.

Two patients experienced spontaneous bleeding of renal angiolipoma during pregnancy (including one who also reported exacerbation of dyspnea). One occurred at week 12 of pregnancy and received left nephrectomy. The other patient had AML bleeding at week 27 of pregnancy and received right lower renal arterial embolization. Both had successful full-term deliveries.

No chylothorax during pregnancy was reported.

### Cases of livebirth in patients taking sirolimus

Six patients had livebirths after taking sirolimus. Detailed information on these factors is listed in Table 2. Half of the 6 patients had livebirths before this pregnancy. The baseline LAM-associated conditions varied greatly among them, including pulmonary functions, St George Respiratory Questionnaire (SGRQ) scores and previous complications. The duration of sirolimus use ranged from 6 to 70 months before pregnancy. Three of them discontinued sirolimus 27, 8, and 12 weeks before pregnancy, and the other 3 patients were taking sirolimus when they were found to be pregnant and then discontinued sirolimus 8, 5 and 4 weeks after pregnancy. Two patients suffered pneumothorax, and one experienced rupture of her AML during pregnancy. None of them had any obstetric diseases. Although two patients reported exacerbated dyspnea after pregnancy compared with before pregnancy, their SGRQ scores did not show significant worsening of quality of life. Seven infants, including a twin, were born without visible malformations despite one of them being admitted to the neonatal intensive care unit for aspiration pneumonia.

**Table 2:** Patients with LAM and a history of parturition after taking sirolimus

Patient no.	1	2	3	4	5	6
Diagnosis	S-LAM	S-LAM	S-LAM	S-LAM	S-LAM	S-LAM
Age of diagnosis	29	30	25	34	26	30
Previous pregnancy	G1P1	G1P0	G1P1	G1P1	G0P0	G0P0
Baseline						
CT grade	III	III	III	NA	NA	II
VEGF-D (pg/ml)	2143.4	1089.4	1591.5	2059.7	NA	4884.4
SGRQ total	76	15	38	14	NA	26
FEV_1_ (L)	0.52	2.50	0.63	1.88	NA	2.22
FEV_1_/Pred (%)	17.4	90.9	21.8	73.9	NA	73.1
FVC (L)	2.21	3.37	1.46	3.44	NA	2.86
FVC/Pred (%)	63.9	106.3	43.9	117.6	NA	81.7
FEV_1_/FVC (%)	23.6	74.1	43.2	54.6	NA	77.7
DLco (mmol/min/kPa)	1.22	4.80	1.26	2.84	NA	4.20
DLco/Pred (%)	13.5	55.3	14.1	33.2	NA	46.5
PaO_2_ (mmHg)	62.9	76.9	70.9	82.5	NA	NA
6MWD (m)	383	675	366	513	NA	520
Complications	None	AML	PTX	CTX	PTX/AML/CTX	PTX
Sirolimus						
Duration (months)	53	46	70	29	12	6
Dose (mg/day)	1.5	1.5	1.0	1.0	1.0	1.0
Serum level (ng/ml)	7.8	7.0	NA	6.0	NA	NA
Time of discontinuation (week)^a^	+ 8	+ 5	+ 4	− 27	− 8	− 12
Pregnancy						
Age of pregnancy (years)	34	34	35	37	27	33
Mode of delivery	C/S	C/S	C/S	VD	VD	C/S
Gestational week	37	35	38	39	38	37
Exacerbated dyspnea	Yes	Yes	Yes	No	No	Yes
Oxygen inhalation (h/day)	24	2–5	1	1	None	1
Complications of LAM	None	Rupture of AML	None	None	PTX	PTX
Obstetric diseases	None	None	None	None	None	None
After parturition						
Exacerbated dyspnea	No	No	No	No	Yes	Yes
Score of SGRQ	56	16	NA	23	NA	32
Sirolimus restart (weeks after delivery)	6	8	4	No	No	24
Infants						
Birth weight (g)	3000	2100/1850^b^	2600	2850	3500	2860
Malformations	No	No	No	No	No	No
NICU experience	No	No	No	No	Yes^c^	No

*AML* angiolipoma, *CTX* chylothorax, *C/S* cesarean section, *DLco* diffusing capacity for carbon monoxide, *FEV*_*1*_ forced expiratory volume in 1 s, *FVC* forced vital capacity, *NICU* newborn intensive care unit, *P*_*a*_*O*_*2*_ partial pressure of oxygen in arterial blood, *PTX* pneumothorax, *SGRQ* St. George’s Respiratory Questionnaires, *S-LAM* sporadic lymphangioleiomyomatosis, *VD* vaginal delivery, *VEGF-D* vascular endothelial growth factor-D, *6MWD* 6-min walking distance

^a^‘+’ means time after pregnancy; ‘−’ means time before pregnancy

^b^This patient delivered twins

^c^The baby was sent to the NICU for aspiration pneumonia and recovered in one week

### Pregnancy in patients with TSC-LAM

Three patients included in this study had TSC-LAM. Two of them had livebirths before diagnosis. Three patients with TSC had 4 pregnancies after the diagnosis of TSC-LAM. Three pregnancies of 2 patients ended in induced abortion in the first trimester. The other patient had a spontaneous abortion at week 6 of pregnancy, and she was the only one of these 3 patients who took sirolimus before pregnancy.

## Discussion

In the current research, we described 30 patients with 34 pregnancies after the diagnosis of LAM. Among these pregnancies, only 10/34 (29.4%) successful livebirths were reported. This contrasts with the 20/32 (62.5%) livebirths in the same group before the diagnosis of LAM. A high rate of spontaneous abortion occurred (6/34), 5 in patients who were on sirolimus when they were pregnant. A total of 18/34 (52.9%) patients selected induced abortion after the diagnosis of LAM. Six patients who took sirolimus before pregnancy reported no abnormalities of their babies.

LAM is a rare cystic lung disease mainly diagnosed in women of child-bearing age [1]. Pregnancy is one of their major concerns when patients are diagnosed with LAM. In a registry study including 230 cases of LAM, 2/3 patients had an experience of pregnancy; 66.9% resulted in livebirth, while spontaneous abortion, therapeutic abortion and still birth were outcomes for 16.7%, 15.0% and 1.4% pregnancies, respectively [7]. Approximately 25% reported worsening respiratory symptoms during pregnancy. Information on pregnancy after the diagnosis of LAM was not described in the study. In a survey of 328 patients with LAM, 2/3 had an experience of pregnancy [8]. Favorable outcomes of pregnancy were found before the diagnosis of LAM. However, LAM diagnosis during pregnancy was associated with a higher incidence of pneumothorax and lower pulmonary function. Patients with LAM also have more premature births and miscarriages. Twelve patients in this study had 15 pregnancies after the diagnosis of LAM. Among these 15 pregnancies after the diagnosis of LAM, 9 resulted in full-term births, and the others included 2 premature births, 1 spontaneous abortion, and 3 induced abortions. Four patients experienced pneumothorax during pregnancy. One patient reported worsening of dyspnea. In a recent study comparing pulmonary function and lung cysts before and after pregnancy in 16 patients, decreased pulmonary function was observed [9].

Considering the potential involvement of estrogen in the pathogenesis of LAM and the risks of disease progression and abnormal pregnancy outcomes, patients with LAM are advised to be cautious regarding pregnancy [5]. Risks include (1) pneumothorax, chylothorax and bleeding from AML; (2) disease progression during pregnancy; and (3) severe dyspnea or hypoxemia for those with lower baseline pulmonary function or during complications. For these reasons, the number of pregnancies decreased greatly in women after the diagnosis of LAM. In the report from Cohen et al., 346 patients had a history of pregnancy before diagnosis, 78.8% of which resulted in full-term births. In contrast, only 30 pregnancies were reported during or after the diagnosis of LAM [8]. However, the prediction model of pregnancy in patients with LAM has not been investigated.

Exacerbated dyspnea, pneumothorax, kidney tumors, or retroperitoneal masses during pregnancy may be the first symptoms of patients with LAM [10–12] [13]. These patients have been described as previously healthy or with a history of asthma or kidney tumors or retroperitoneal mass that had not been noticed before. For any woman during pregnancy, an unexplained degree of dyspnea, pneumothorax, kidney tumors, or retroperitoneal mass, a diagnosis of LAM or TSC should be listed in the differential diagnosis.

Pregnant women with LAM have an increased risk of complications. The risk factors should be evaluated carefully for every patient who is diagnosed with LAM. Evaluation should include (1) the degree of LAM on high-resolution CT, (2) baseline pulmonary function and pulmonary function decline rate, (3) previous history of pneumothorax or chylothorax, (4) existence and sizes of kidney AML or retroperitoneal lymphangioleiomyomas, (5) TSC, (6) sirolimus treatment, and (7) previous history of pregnancy. A multidisciplinary team including obstetricians, pulmonary physicians, thoracic surgeons, urologists, interventional radiologists, and geneticists is recommended to ensure optimal management during pregnancy [1].

Pulmonary function decline is common during pregnancy. In an analysis of a Japanese cohort, 10 pregnant women experienced a rapid decline (n = 1) or decline (n = 9) in pulmonary function [14]. In an observation of 4 cases, serum VEGF-D did not change much during pregnancy [15]. VEGF-D is a biomarker reflecting the disease severity of LAM [16].

Sirolimus is commonly used in the treatment of LAM. It is advised to discontinue sirolimus 12 weeks before pregnancy, during the time of pregnancy and while breastfeeding [1]. In this study, sirolimus was related to high spontaneous abortion. However, favorable outcomes were seen in 6 successful pregnancies. Among the 6 patients who had full-term livebirths, 3 patients stopped sirolimus 8–27 weeks before pregnancy, and 3 patients stopped sirolimus 4–8 weeks during pregnancy. No obvious abnormalities were found in the babies whose mothers were pregnant while on sirolimus. Sirolimus was labeled Pregnancy Category C and was only considered for use in pregnancy when the potential benefit was judged to outweigh the potential risks to the embryo or fetus.

Patients with LAM may experience exacerbation of dyspnea, hypoxemia, or rapid decline of pulmonary function when they discontinue sirolimus during pregnancy. In a case report, sirolimus was successfully used intermittently in a patient’s first pregnancy and continuously in her second pregnancy [17, 18]. Pulmonary function was maintained well even at a reduced dose during her pregnancy. Whether low-dose sirolimus could be used for pregnant women with moderately or severely impaired pulmonary function is still unknown. Regardless, it seems that an unplanned pregnancy during sirolimus treatment could safely continue after discontinuing sirolimus.

Management of pneumothorax depends on the degree and stage of progression. A chest tube is commonly used with or without suction. For patients with hemodynamically unstable bleeding of AML, emergency arterial embolization or surgery is required [19]. If patients are hemodynamically stable, conservative management is preferred [20].

There are some limitations of this report. The first is low response rate to this study. In the early version of the protocol of our registry, information on pregnancy was incomplete. Additionally, symptoms, oxygen, and pulmonary function during and after pregnancy were missing. Detailed information should be incorporated into the future protocol of the LAM registry.

In summary, patients with LAM may have increased risks of disease progression, complications, and abnormal outcomes of pregnancy. Careful evaluation regarding the disease severity, complication possibilities, and potential genetic disease associated with TSC should be performed before planning a pregnancy. While sirolimus was likely associated with a high risk of spontaneous abortion, for patients on sirolimus who were pregnant, sirolimus did not produce worse outcomes in our limited observation.

## Data Availability

The dataset used in this research and analysis were available from the corresponding authors.

## Abbreviations

AML: Angiolipoma
ATS: American Thoracic Society
C/S: Caesarean section
CT: Computed tomography
CTX: Chylothorax
DLco: Diffusing capacity for carbon monoxide
ERS: European Respiratory Society
FEV_1_: Forced expiratory volume in 1 s
FVC: Forced vital capacity
JRS: Japanese Respiratory Society
LAM: Lymphangioleiomyomatosis
NICU: Newborn intensive care unit
P_a_O_2_: Partial pressure of oxygen in arterial blood
PTX: Pneumothorax
SGRQ: St. George’s Respiratory Questionnaires
S-LAM: Sporadic lymphangioleiomyomatosis
TSC: Tuberous sclerosis complex
VD: Vaginal delivery
VEGF-D: Vascular endothelial growth factor-D
6MWD: 6-Min walking distance
